# Child development beyond the nutrition-specific models: bridging the pathways via psychosocial stimulation

**DOI:** 10.3389/fpsyg.2023.1273591

**Published:** 2023-11-23

**Authors:** Blessy E. David, Sanjay Kumar

**Affiliations:** ^1^Thapar School of Liberal Arts and Sciences, Thapar University, Patiala, Punjab, India; ^2^Department of Psychology, School of Arts, University of Allahabad, Prayagraj, Uttar Pradesh, India

**Keywords:** malnutrition, psychosocial stimulation, child development, developmental delays, nutrition-sensitive intervention

## Abstract

**Background:**

Undernutrition has severe and lasting consequences on child development. Evidently, the interventions based on the biomedical model with food and direct supplementation have failed to produce the desired outcomes for decades. In light of the established positive effects of psychosocial stimulation on developmental outcomes, we propose that the observed failures relate to not including psychosocial stimulation within the interventions. Here we test whether psychosocial stimulation mediates the association between nutritional status and motor and mental development in a large cohort from Madhya Pradesh, India.

**Method:**

Using a correlational design in children below 3 years of age (*N* = 275; Males = 148, Females = 127) nutritional status was measured through mid-upper arm circumference (MUAC), psychosocial stimulation was assessed with the HOME-inventory, and motor and mental development were assessed with the Developmental Assessment Scales for Indian Infants (DASII). We assessed the effectiveness of 25-week intervention model incorporating psychosocial stimulation on 95 undernourished children in experimental group and 77 in control group.

**Results:**

The study found that psychosocial stimulation fully mediated the relationship between nutritional status and the motor development [Effect = 1.03, 95% C.I. (0.19, 2.04), *p* = 0.05] and mental development [Effect = 0.49, 95% C.I. (0.09, 1.03), *p* = 0.05] in children under 3 years of age. Nutritional status significantly predicted the psychosocial stimulation to the child (*R*^2^ = 0.77). Analyzing the effectiveness of the intervention program revealed significant reduction in the developmental delays in both the motor [*t*(81) = 2.568, *p =* 0.012] and mental development [*t*(81) = 4.506, *p =* 0.001] of the undernourished children.

**Conclusion:**

Findings indicate that nutrition translates into positive developmental outcomes in a child only with the scaffolding effects of psychosocial stimulation primarily received from home. Integrating psychosocial stimulation activities like storytelling, play, art and crafts, puppets, travel etc. in the intervention programs designed to address undernutrition may yield rich dividends in bridging the developmental delays among undernourished children.

## Introduction

Undernutrition is viewed as a health outcome as well as a risk factor resulting from inadequate diet and disease. It is a global concern as it makes a child an easy prey to infection and delayed recovery (Blössner et al., 2005). Thus a potentially lethal cycle of worsening illness and deteriorating nutritional status is created (UNICEF, 2007). It also carries the threat of intergenerational transmission since an undernourished mother is likely to bear a low birth-weight child eventually susceptible to disease and premature death (Blössner et al., 2005).

### Negative impact of undernutrition on child development

Studies report that the first 1,000 days of a child’s life beginning from conception are crucial concerning the development of cognitive abilities, motor abilities, and work performance throughout life (Aboud and Yousafzai, 2015). This period is also vital for brain development through neurogenesis, axonal, and dendritic growth, synaptogenesis, cell death, synaptic pruning, myelination, and gliogenesis (Walker et al., 1991). The negative developmental impact of undernutrition at this stage is reported in dysfunctions of the executive system, affecting the brain’s structural and functional capacity lifelong (Selvam et al., 2018). Undernutrition also has several far-fetched consequences like- vulnerability to physical and mental illnesses, apathy, poor attention, underperformance in schools, less positive affect, and difficulty in forming healthy relationships (Walker et al., 2007; Black et al., 2013; McGrath and Schafer, 2014). In this way, undernutrition prevents millions of children across the globe from reaching their apex developmental potentials throughout life (Georgieff, 2007). A study exploring the relevance of plyometric program among children and adolescents with ADHD reports positive relationship between cognitive functions, aerobic fitness tests and motor skills (Hattabi et al., 2021).

### Inadequacy of the intervention programs for undernutrition

The problem of undernutrition has been identified as a global concern among the 17 sustainable development goals (SDGs) proposed by the UN in 2015 (UNHCR, 2017). The World Health Organization (WHO), United Nations International Children’s Emergency Fund (UNICEF), World Bank, and various other national agencies are striving their level best to eradicate the problem (Hoddinott et al., 2008). The intervention programs have predominantly relied on the nutrition-specific strategies focused primarily on the immediate and explicit factors associated with child nutrition and development (UNICEF, 2007; Government of India, 2011). The programs have been limited to the inclusion of Vitamin-A and zinc supplementation, promoting breastfeeding practices, distribution of food packets, dietary diversity promotion, and food fortification (Achadi et al., 2016). Reportedly, over the decades these intervention programs have had a dismal influence in improving the health status and bringing about the overall development in an undernourished child (Dixit et al., 2018).

Hence, the marginal success rates challenge the conventional understanding that a child’s development is a single factor phenomenon contingent upon nutrition solely. Researchers have shown that several psychosocial determinants of child development like- water, hygiene, child protection, schooling, mother’s mental health, social safety, women empowerment, etc. are not adequately addressed in the designing of the intervention programs (Ruel and Alderman, 2013). In its review of the Global Nutrition Policies ongoing in 123 countries and territories, WHO (2013) reaffirms the need to incorporate the nutrition-sensitive paradigm for the comprehensive development of the child. The nutrition-sensitive intervention paradigms incorporate the implicit psychological and social determinants along with nutrition to bring about holistic child development (Nahar et al., 2012). However, the question as to how the psychosocial factors mediate the association of nutrition and child development remains unanswered (Aboud and Yousafzai, 2015). The present study is an attempt to bridge this existing gap in the literature by assessing the mediating influence of psychosocial stimulations on child development.

### Psychosocial stimulation as a mediating factor in determining child development

Studies report that the undernourished child is usually characterized by inactivity, apathy, and unresponsiveness to the environment (Walker et al., 1991; McGrath and Schafer, 2014). On the contrary, a healthy developing child is capable of self-exploration and actively operates upon the various psychological and social stimulations available in the environment (Walker et al., 2007). Researches have established that psychosocial stimulations help to fine-tune and strengthen the neural pathways within the developing brain (Engle et al., 2007; McGrath and Schafer, 2014). When the psychosocial stimulation is compromised as in the case of the undernourished child, it eventually hinders the development of the young brain to its full capacity (Rahman et al., 2004; McGrath and Schafer, 2014; Dabar et al., 2016). Therefore, in the present study, we hypothesize that:

*H1:* the child’s nutritional status of being healthy or undernourished is positively associated with the level of psychosocial stimulation received by the child.

A developing child receives psychosocial stimulations primarily through responsive parenting practices, expressions of warmth and love, conscious affirmations to the child, interactive play, and mother singing or talking to the child (UNICEF, 2012). The father’s expressions of encouragement, attention, smile, touch, helping a child explore the outside world, etc. are also reportedly the rich sources of psychosocial stimulation to the developing child (UNICEF, 2012; Ruel and Alderman, 2013). In this way, the parents and the home environment they provide to the child are key to psychosocial stimulations for the adequate development of the child. However, in circumstances of economic hardships, maternal depression, domestic violence, conflicts within the home, etc. the availability of psychosocial stimulation to the child are found severely compromised (Murray et al., 1996). When undernutrition couples with unstimulating home environments, it leaves lasting negative impacts on the child’s cognitive, motor, socio-emotional, and language development (Raver and Knitze, 2002; Grantham-McGregor et al., 2007). Studies have also shown that motor-demanding physical exercise has positive effects on the executive function of children with developmental coordination disorders (Tajari et al., 2023). Therefore, it was hypothesized that:

*H2:* Psychosocial stimulation received by the child at home through positive parenting and family engagement practices is positively correlated to the child’s motor and mental development.

*H3:* The child’s nutritional status and psychosocial stimulation received at home would together predict the child’s motor and mental development significantly.

Therefore, an effective intervention program for the undernourished children should involve exposing a child to positive stimulating experiences at home along with supplementation of nutrition to achieve wholesome development. Intervention programs incorporating psychosocial determinants have shown a significant impact even in critical situations and emergencies like- the food crisis in the Sahel in West Africa (McGrath and Schafer, 2014). Similar pathways between nutrition, stimulation, and development have been explored before by Larson et al. (2018).

*H4:* The repeated measures t-test analysis following the 25-week intervention program would show significant improvement in the motor and mental development of children.

The theoretical framework for the present study is based on the ecological systems theory proposed by Bronfenbrenner and Morris (2007). The theory proposes that a child’s development takes place in multiple contexts of ecological systems, eventually supporting or stifling it. The first among the five interlocking contextual systems is the microsystem which is the sphere of immediate external influence on a growing child involving relationships with parents, siblings, relatives, and society. This is the stage where the foundations of development are laid in the growing child through the day-to-day interactions in the immediate social context also referred to as the psychosocial stimulations in the present study. Similarly, mesosystem, exosystem, macrosystem, and chronosystem are the other sources of psychosocial stimulation to the child through their interactions with peers, neighbors, social groups, political systems, and culture, etc. In the present study, psychosocial stimulations received by the child are measured using the Home Observation for Measurement of the Environment (HOME) inventory originally developed by Bradley (1993). It was designed to measure the quantity and quality of stimulation and support available to a child in the home environment. The scale includes items reporting parental responsivity, acceptance of the child, organization of the environment, learning materials, parental involvement, and variety within the home environment.

## The present study

The present study examined the correlations among a child’s nutritional status, psychosocial stimulation, and child development, particularly in the Indian context. The nutritional status was measured as the mid-upper arm circumference (MUAC) on a continuous scale (WHO, UNICEF, 2009). Psychosocial stimulations were measured by the Home Observation for Measurement of the Environment (HOME) inventory (Bradley, 1993). The child’s motor and mental development quotients were computed from the performance-based psychometric test called Developmental Assessment Scales for Indian Infants (DASII) (Phatak, 1997). It is the Indian adaptation of Bayley Scales of Infant Development (BSID) (Bayley, 1969). The study aims to explore the mediating role of psychosocial stimulation in predicting the relationship between nutrition and development in preschool children through simple mediation analysis. The tested mediation model accounts for the parental education status, father’s occupation, family income, and the place of residence as the covariates in the study. Following the baseline assessments of psychosocial stimulation and measures of development, the undernourished children were divided into experimental group receiving psychosocial stimulation-based intervention along with food-intervention and control group receiving food-based intervention alone for 25-weeks.

## Method

### Research design and sample

The study applied the correlational design followed by the simple mediation analysis to interpret the findings. The effectiveness of the intervention program was analyzed using the repeated measures t-test analysis. The state of Madhya Pradesh, located in central India is rated high among the states bearing the most burden of child undernutrition. Notably, the Sagar district of Madhya Pradesh falls in high prevalence category as it shares 41.00% of the total cases of stunting reported within the state (NFHS, 2016). The present study is conducted in the Sagar district of Madhya Pradesh situated on the Vindhya Range 1,758 feet (536 m) above sea-level. The city is around 172 kilometers (107 mi) northeast of the state capital, Bhopal (Krishnan, 1967), and also hosts a central university funded by the government of India. Among the 11 sub-divisions of the Sagar district, the Sagar sub-division, as well as the Khurai sub-division, were randomly selected through the lottery method.

In further shortlisting, the villages or wards for the recruitment of research participants, we also considered the availability of stimulations to the child in the form of education, health-care, transport, proximity to the market, etc. Thus, altogether 27 locations (Sagar-6 Aanganwadi centers, Kurai- 9 wards and 12 villages) were shortlisted for data collection by picking up chits randomly out of the total 53 sites. In this way, we obtained a list of altogether 653 children (Sagar-254, Khurai-399) who were below the age of 3 years from the records of the state government-run Aanganwadi centers and the internationally acclaimed non-governmental organization (NGO)-World Vision India.

Given the applied inclusion and exclusion criteria, finally, a sample of 275 children (Sagar-103, Khurai-172) was obtained. Among them, 103 were healthy (*M* = 58, *F* = 45), and 172 were malnourished (*M* = 90, *F* = 82) in the age range 05.20 to 33.40 months (Mean = 17.64). About half of the children in the sample (50.18%) were firstborn (*N* = 138) while, 36.73% were born second (*N* = 101) among the siblings in their homes, and the rest (13.09%) were either born third or lower in the order. Participants predominantly hailed from joint families (*N* = 220), which is a form of an extended family consisting of parents, their children, spouses of the children, and their offspring in one household as compared to nuclear family set-up (*N* = 55) where only parents and their children cohabit a place. Since the child’s home is the primary source of psychosocial stimulation, the extended family system opens ample avenues for the child to receive psychosocial stimulation as compared to the nuclear (Small) family structures.

#### Inclusion criteria

The Study included children below the age of 3 years as the research participants. We also included singletons, twins, healthy (MUAC > 115 mm), malnourished (MUAC < 115 mm) as well as children born low in birth weight (<2.5 Kg).

#### Exclusion criteria

We excluded children from the final sample on the following grounds- the child/mother was out of station during the visit (112), children were sleepy/uncooperative during the study (85), were ill/hospitalized in the 2 weeks before the appointed visit (78). Some mothers refused to participate in the study (59), children had other physical/mental handicaps (11), and the ones raised by a single parent (33). Studies have documented that single-parent status is a risk factor for poor developmental outcomes, which is the reason why children raised by a single parent were excluded from the study to avoid the possible confounds (Raver and Knitze, 2002).

### Procedure

The Institutional Human Ethics Review Board (IHERB) approved the present study. We approached the participants through the supervisors at the state government-run Aanganwardi centers, health workers as well as volunteers employed in World Vision India involved in the respective areas under investigation. Following the screening, each child was visited at the respective home individually by a team comprising of a faculty supervisor from the university and a doctoral student trained in the administration, scoring, and interpretation of the performance measure of child development- Developmental Assessment Scales of Indian Infants (DASII). The team was also comprised of two post-graduate university students trained in interview and observation skills. The same group of a faculty supervisor, doctoral student, and post-graduate students continued throughout the study for each visit. Further, we obtained the written informed consent of the mother/primary caregiver of the child to administer the performance test of developmental delay (DASII) on the child. The average administration time for each participant was 25–30 min, including the administration of the performance measure of developmental delay (DASII), parent-reported measure for psychosocial stimulation, and the physical measurements. The 172 undernourished children were randomly divided into two groups- Experimental group (*N* = 95) receiving the psychosocial stimulation-based intervention along with food-based intervention and Control group (*N* = 77) receiving only the food-based intervention. Both the groups were observed for the next 25-weeks to assess the effectiveness of the intervention program.

#### Intervention program

The 25-week intervention program comprised of both the experimental and control group children receiving food-based intervention. This included weekly take-home ration, food baskets, diet-plans and Timely targeted counseling (TTC) for the mothers. In addition to this, the experimental group received psychosocial stimulation-based intervention. This included intervention kit for each child comprising of play items, puzzles, art and craft workbook, colors etc. The research team and the NGO volunteer would visit the children in their respective homes every week and educate the mothers on the importance of play, touch, asking questions, reading and narrating stories, singing songs to the child, taking the child to travel and allowing the child to explore the nature. Such activities provide the malnourished child with the much-needed psychosocial stimulation to bridge the developmental delays. Both the groups were assessed for the motor and mental development again following the 25-week intervention program.

Phase-wise plan of the research depicting the sample distribution and the assessments and treatments administered 
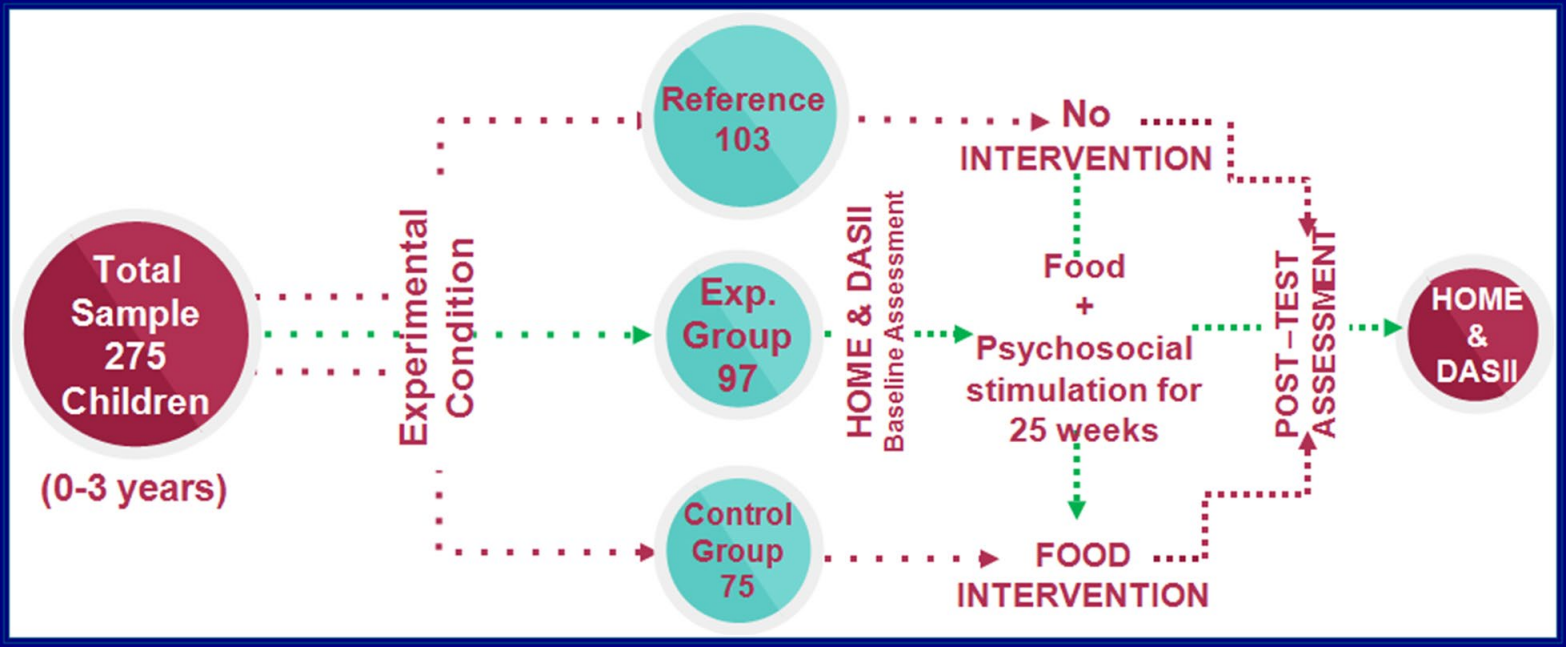


### Measures in the study

#### Predictor

##### Mid-upper arm circumference

The nutritional status of the child was the predictor variable in the tested mediation model. It was measured on a continuous scale as the mid-upper arm circumference (MUAC) measure of each child. Theoretically, the MUAC of 115 mm and weight-height *Z*-score of −3 S.D. has been marked as indicators for severe undernutrition as recommended by WHO, UNICEF (2009).

#### Outcome measures

##### Developmental assessment scales for Indian infant

The motor developmental quotients (MoDQ) and the mental developmental quotients (MeDQ) were the two outcome measures in the tested mediation model. They were measured using the performance-based Developmental Assessment Scales for Indian Infants (DASII) originally developed by Pathak (1970). It is the Indian Adaptation of the Bayley Scales of Infant Development (BSID) (Bayley, 1969). The present research employed the revised version of DASII as a performance-based assessment tool for the developmental delays in children below the age of 3 years (Phatak, 1997). The scale covers two significant domains- motor development (67 items; α = 0.88) and mental development (163 items; α = 0.91). The items are administered serially and scored as pass/fail based on the child’s performance. The motor development items cover body balance, basic locomotive skills, reaching, picking, and other manipulatory behavior. Similarly, the mental development items record the child’s cognizance, perceptual pursuit, exploration, development of communication and language comprehension, spatial relationship, manual dexterity, imitative behavior, social interaction, etc. We terminate the scoring at the particular item where the child records five consecutive failures. The median reliability index for motor and mental scales based on correlations between consecutive months is reported to be 0.88 for motor scale and 0.91 for mental scales.

#### Mediator

##### Home observation for measurement of the environment

Psychosocial stimulation available to the child was the mediating variable in the tested mediation model. It was measured using the Home Observation for Measurement of the Environment (HOME) inventory initially developed in the English language by Bradley (1993). The translation into simple Hindi language, as well as the Indian adaptation of the HOME inventory, was done by Kohli et al. (2005). The scale (35 items; α = 0.94) is scored as yes/no based on the mother’s response to each item. A ‘yes’ is scored as 1 and a ‘no’ response is scored as a 0 value which eventually leads to a summated domain score. Split half reliability (after correction for length by Spearman-Brown prophecy) was ascertained to be 0.41, the index of reliability was0.64 and the relative reliability was 0.41. The scale is classified into four major domains. The first one is the home environment which includes items describing the child’s home, cleanliness, and the availability of play items at home. The second domain is Play and language motivation involving items describing the use of language to communicate with the child, the efforts to facilitate language development in the child, availability of play areas for the child to explore, etc. The third domain is the mother’s affection and affirmations to the child which includes items describing the mother’s acts of displaying affection and warmth to the child. The fourth domain is travel and exploration entailing items describing the availability of opportunities to grow and learn from educational and recreational tours, audio-visual stimulation, play, and craft, etc.

#### Demographic covariates

Parental education status as reported by the father and mother, father’s occupation type, the family’s annual income as reported in Indian rupees, and the type of residence were treated as the covariates in the tested mediation model. Type of residence refers to whether the child hails from the rural, semi-urban, or urban place of residence. Each variable mentioned as covariate influences the quality and quantity of psychosocial stimulation available to the child in the form of available resources, time spent with the child, etc.

## Statistical analysis approach

The primary variables under consideration were nutritional status (MUAC) and psychosocial stimulation (HOME) predicting the Motor development (MoDQ) and the mental development (MeDQ) of the preschool children. Preliminary analyses using SPSS (v22.0, IBM) tested the predictors and criterion variable for normality of distribution and outliers. The correlations between the variables under study, namely the nutritional status (MUAC), psychosocial stimulation (HOME), measures of child development (MoDQ and MeDQ), and the demographic variables were measured. It was followed by the simple mediation analysis, which was proposed by Baron and Kenny (1986). Here, we examined the association between the nutritional status of the child (MUAC) and the measures of child development (MoDQ and MeDQ) when psychosocial stimulation (HOME) intervened as the mediating variable. The parental education status, father’s occupation, family’s annual income, and residence were treated as covariates in the proposed model.

**Figure 1:**
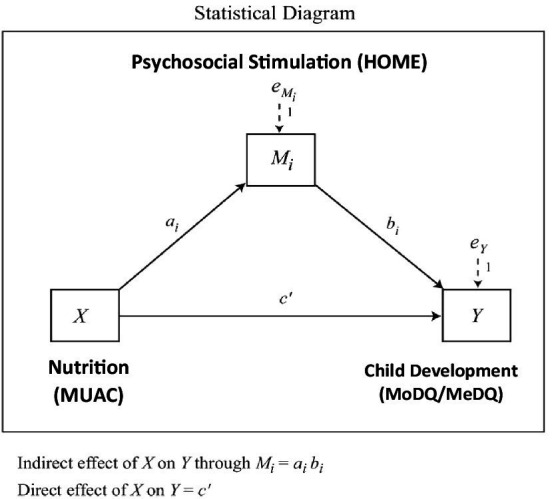
Proposed model for the study.

The Figure 1 illustrates the mediational relationship as tested in the present study. Psychosocial stimulation (HOME) could be considered a mediator if (a) Nutritional status (MUAC) significantly predicts child development (MoDQ and MeDQ) via psychosocial stimulation (HOME); (b) Nutritional status (MUAC) significantly predicts Psychosocial stimulation (HOME); and (c) Psychosocial stimulation (HOME) significantly predicts child development (MoDQ and MeDQ), having controlled for nutritional status (MUAC). ‘Perfect’ mediation could be said to have occurred where no significant association remains between nutritional status (MUAC) and child development (MoDQ and MeDQ), once psychosocial stimulation (HOME) is controlled. For mediation analysis to be appropriate, we should meet two conditions. First, MUAC and HOME must share the anticipated association. Second, the MoDQ and MeDQ would need to be significantly associated with both MUAC and HOME. The path analysis was done using the statistical package Process 3.1 (Model 4) developed by Hayes (2017).

A separate mediation analysis was conducted to further explore the varying predictions of each domain of psychosocial stimulation on the developmental outcomes. A domain total was first computed by adding scores for items representing a particular domain of psychosocial stimulation in the HOME inventory. In this way, we had scores representing the four domains of psychosocial stimulation namely-home environment, language and play, mother’s affection and affirmation, travel and exploration. The simple mediation analysis (model 4) was repeated by substituting each domain score as a mediator separately for the path analysis. In the present study, the mediational paths were also tested separately for the group of healthy and undernourished children.

## Results

### Descriptive statistics and intercorrelations

Tables 1–3 report the descriptive statistics of the variables in the study. Table 4 reports intercorrelations among the variables. Nutrition and psychosocial stimulation were significantly associated with each other (*r =* 0.369, *p* ≤ 0.01) as well. Psychosocial stimulation is significantly related to other determining factors of a child’s development- family income, father’s education, father’s occupation, and mother’s education. The child’s residence and the psychosocial stimulation are also significantly associated with each other (*r =* 0.848, *p* ≤ 0.01). These results provide support for the hypotheses related to the variable relationships.

**Table 1:** Sociodemographic characteristics of participants at baseline with reference to gender, birth order, residence, type of house, and type of family.

Baseline characteristic	Healthy		Undernourished			
	Reference group		Experimental group		Control group	
Total participants (*N* = 275)	*N* = 103		*N* = 97		*N* = 75	
	*n*	*%*	*n*	*%*	*n*	*%*
Gender						
Male	58	56.30	52	53.60	38	50.70
Female	45	43.7	45	46.40	37	49.30
Birth order						
First	56	54.40	44	45.40	38	50.70
Second	39	37.90	35	36.10	27	36.00
Third and higher	08	7.80	18	18.50	10	13.30
Residence						
City (municipal corporation)	103	100.0	00	00	00	00
Ward	00	00	37	38.10	39	52.00
Village (Panchayat)	00	00	60	68.90	36	48.00
Type of house						
Pakka house (Concrete-house)	102	99.00	48	49.50	39	52.00
Semi-Pakka (concrete/mud)	01	1.00	13	13.40	08	10.70
Kaccha (Mud-house)	00	00	36	37.10	28	37.30
Type of family						
Joint	77	74.80	79	81.40	64	85.30
Nuclear	23	25.20	18	18.60	11	14.70

**Table 2:** Sociodemographic characteristics for the continuous variables.

Baseline characteristic	Min	Max	Mean	S.D.
Child’s age in months	05.20	33.40	17.64	06.480
Child’ height in cm	55.00	94.00	75.00	07.301
Child’ weight in kg	04.00	14.50	08.77	01.650
Birth weight in kg	01.00	04.50	02.58	00.570
Mother’s age in years	19.00	46.00	26.44	04.797
Years of marriage	01.00	35.00	05.96	04.132

**Table 3:** Descriptive statistics for the measure of nutritional status (MUAC) and the dependent measures of the study (HOME, MoDQ, MeDQ).

Variable	Min	Max	Mean	S.D.	Skewness	Kurtosis
Developmental motor quotient (MoDQ)						
Reference group	56.50	166.83	105.72	22.23	0.611	0.289
Experimental	46.94	154.73	102.87	23.96	–0.109	–0.462
Control	37.84	158.78	89.92	28.75	0.100	–0.872
Developmental mental quotient (MeDQ)						
Reference group	56.91	120.00	84.63	14.29	0.192	–0.264
Experimental	39.60	119.35	81.33	15.74	0.144	–0.039
Control	39.63	117.86	72.94	17.49	0.471	–0.274
Psychosocial stimulation (HOME)						
Reference Group	10.00	31.00	22.19	4.29	–0.520	–0.082
Experimental	1.00	13.00	7.60	2.66	–0.141	–0.718
Control	1.00	13.00	7.05	2.91	0.060	–0.454
Nutritional status (MUAC)						
Reference Group	11.50	15.00	13.63	0.86	–0.130	–0.288
Experimental	11.20	15.00	12.78	0.85	0.369	–0.499
Control	11.50	15.00	12.93	1.07	0.444	–0.825

MUAC, mid-upper arm circumference measure, HOME is the measure of the available psychosocial stimulation; MoDQ, motor development quotient; MeDQ, mental development quotient.

**Table 4:** Intercorrelations and descriptive statistics.

Variables	*M*	SD	1	2	3	4	5	6	7	8	9	10	11	12	13
1. MUAC	13.14	0.99	--												
2. HOME total	12.91	7.96	0.369**	--											
3. MoDQ	100.40	25.52	0.154*	0.270**	--										
4. MeDQ	80.28	16.37	0.145*	0.257**	0.433**	--									
5. Annual income	2.05	2.22	0.185**	0.375**	0.044	0.017	--								
6. Father’s occupation	--	--	0.076	0.349**	0.056	0.036	0.177**	--							
7. Mother’s education	--	--	0.364**	0.668**	0.155*	0.160**	0.379**	0.375**	--						
8. Father’s education	--	--	0.327**	0.605**	0.173**	0.211**	0.356**	0.230**	0.721**	--					
9. Residence	--	--	0.295**	0.848**	0.136*	0.162**	0.307**	0.408**	0.603**	0.528**	--				
10. HOME environment	2.72	2.67	0.336**	0.855**	0.197**	0.215**	0.337**	0.245**	0.542**	0.488**	0.660**	--			
11. HOME language/Play	3.21	2.20	0.341**	0.901**	0.254**	0.236**	0.342**	0.285**	0.674**	0.599**	0.760**	0.692**	--		
12. HOME affection	3.25	2.37	0.361**	0.928**	0.214**	0.239**	0.317**	0.372**	0.625**	0.558**	0.857**	0.718**	0.786**	--	
13. HOME travel/explore	3.71	1.87	0.229**	0.809**	0.302**	0.215**	0.318**	0.328**	0.503**	0.478**	0.702**	0.497**	0.692**	0.748**	--

Significance levels have been denoted as- * Significant at *p <* 0.05 level, ** Significant at *p <* 0.01 level. MUAC, mid-upper arm circumference measure, HOME total-measure of psychosocial stimulation. MoDQ, motor development quotient; MeDQ, mental development quotient. The four Domains of Psychosocial stimulation are shown from 10 to 13 indicating the letters HOME.

### Mediation analysis

The complete test of the hypothesized model was performed using PROCESS macro (Model 4) using SPSS (v22.0, IBM). The model posits the nutritional status of the child (MUAC) as the predictor variable, psychosocial stimulation (HOME) as the mediating factor, and the child’s motor developmental quotient (MoDQ) and mental developmental quotient (MeDQ) as the dependent measures of the study. The family’s annual income, parental educational status, father’s occupation, and the child’s residence were treated as covariates in the model. The results of the mediation analysis are reported in Table 5.

**Table 5:** Results of mediation analysis combined for both the healthy and undernourished group of children

	Mediator				Dependent variables							
	Psychosocial stimulation (HOME)				Motor development (MoDQ)				Mental development (MeDQ)			
Antecedents	*B*	SE	*t*	*R* ^2^	*B*	SE	*t*	*R* ^2^	*B*	SE	*t*	*R* ^2^
				0.77				0.13				0.10
Constant	12.93	3.41	3.41***		37.21	22.12	1.68		48.81	14.41	3.39***	
Psychosocial stimulation	--	--	--		1.74	0.39	4.52***		0.84	0.25	3.35***	
Nutritional status (MUAC)	0.59	0.25	2.34**		1.28	1.61	0.79		0.84	1.05	0.80	
Father’s education	0.50	0.21	2.37**		0.599	1.35	0.44		1.42	0.88	1.60	
Mother’s education	0.70	0.23	3.02***		0.15	1.49	0.11		0.69	0.97	0.72	
Father’s occupation	0.17	0.27	0.64		3.32	1.67	1.98*		0.32	1.09	0.30	
Annual income	0.00	0.00	2.14*		0.00	0.00	–1.11		0.00	0.00	–1.68	
Residence	6.29	0.36	17.43***		8.39	3.33	2.52**		3.68	2.17	1.70	

					Effect	SE	LLCI	ULCI	Effect	SE	LLCI	ULCI
Indirect effects of Nutritional status of the child (MUAC) via psychosocial stimulation					1.03	0.46	0.19	2.04	0.49	0.24	0.09	1.03
					Effect	SE	LLCI	ULCI	Effect	SE	LLCI	ULCI
Direct effects of Nutritional status of the child (MUAC)					1.28	1.61	–1.89	4.44	0.83	1.05	–1.22	2.09

LLCI, bias corrected lower limit confidence interval; ULCI, bias corrected upper limit confidence interval. Unstandardized regression coefficients are shown; bootstrap sample size is 5000. Significance levels have been denoted as- * Significant at *p <* 0.05 level, ** Significant at *p <* 0.01 level, *** Significant at *p <* 0.001 level. MUAC, mid-upper arm circumference measure, HOME total-measure of psychosocial stimulation; MoDQ, motor development quotient; MeDQ, mental development quotient.

To test for the first hypothesis (*H1*), the regression path between nutritional status and the psychosocial stimulation was analyzed. The analysis for the path (a) between MUAC and HOME confirms the hypothesis that nutrition significantly predicted psychosocial stimulation [*b* = 0.59, *t*(268) = 2.34, *p* ≤ 0.001]. The model significantly accounted for a 77.35% change in psychosocial stimulation of the child [*F*(1,268) = 152, *p* ≤ 0.001, *R*^2^ = 0.77].

**Figure 2:**
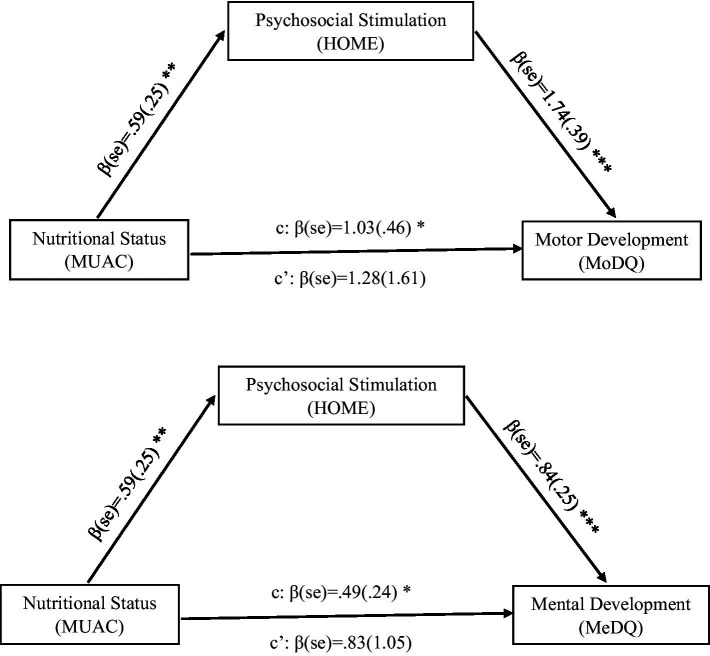
Results of mediation analysis combined for both the healthy and undernourished group of children. Unstandardized regression coefficients for the relationship between nutritional status and developmental outcomes as mediated by psychosocial stimulation. Significance levels have been denoted as- * Significant at *p <* 0.05 level, ** Significant at *p <* 0.01 level, *** Significant at *p <* 0.001 level.

To test for the second hypothesis (*H2*), the regression paths between psychosocial stimulation and the indices of a child’s motor and mental development were analyzed. The analysis for the path (b) confirms the hypothesis that psychosocial stimulation (HOME) significantly predicted the child’s motor development denoted by MoDQ [*b* = 1.74, *t*(267) = 4.52, *p* ≤ 0.001] and mental development denoted by MeDQ [*b* = 0.84, *t*(267) = 3.35, *p* ≤ 0.001]. Here, Psychosocial stimulation accounts for about 12.55% of motor development [*F*(1,267) = 5.47, *p* ≤ 0.001, *R*^2^ = 0.12] and 9.77% of mental development [*F*(1,267) = 04.13, *p* ≤ 0.001, *R*^2^ = 0.10] in the child (Figure 2).

To test for the third hypothesis (*H3*), the combined regression paths between nutritional status, psychosocial stimulation, and the indices of a child’s motor and mental development were analyzed. Overall, the mediation model as depicted by the indirect path (c), significantly predicted the motor development [Effect = 1.03, 95% C.I. (0.19, 2.04)] as well as mental development [Effect = 0.49, 95% C.I. (0.09, 1.03)] in the children. Moreover, the direct path (c’) shows there was no longer a significant association between MUAC and MoDQ/MeDQ, after controlling for HOME. The results depict that child’s psychosocial stimulation perfectly mediates the association between nutritional status and child development (Tables 6–8).

**Table 6:** Results of mediation analysis depicted separately for the healthy group of children.

	Mediator				Dependent variables							
	Psychosocial stimulation (HOME)				Motor development (MoDQ)				Mental development (MeDQ)			
Antecedents	*B*	SE	*t*	*R* ^2^	*B*	SE	*t*	*R* ^2^	*B*	SE	*t*	*R* ^2^
				0.78				0.14				0.14
Constant	17.85	8.26	2.16*		84.58	49.87	1.70		58.60	31.36	1.87*	
Psychosocial stimulation	--	--	--		1.91	0.53	3.60***		1.28	0.33	3.85***	
Nutritional status (MUAC)	0.27	0.58	0.47		–0.84	3.45	–0.24		0.21	2.17	0.10	

					Effect	SE	LLCI	ULCI	Effect	SE	LLCI	ULCI
Indirect effects of Nutritional status of the child (MUAC) via psychosocial stimulation					0.52	1.18	–1.56	3.18	0.34	0.77	–1.08	2.48

					Effect	SE	LLCI	ULCI	Effect	SE	LLCI	ULCI
Direct effects of nutritional status of the child (MUAC)					–0.84	3.45	–7.67	6.00	0.21	2.17	–4.09	4.51

LLCI, bias corrected lower limit confidence interval; ULCI, bias corrected upper limit confidence interval. Unstandardized regression coefficients are shown; bootstrap sample size is 5000. Significance levels have been denoted as- * Significant at *p <* 0.05 level, ** Significant at *p <* 0.01 level, *** Significant at *p <* 0.001 level. MUAC, mid-upper arm circumference measure, HOME total-measure of psychosocial stimulation; MoDQ, motor development quotient; MeDQ, mental development quotient.

**Table 7:** Results of mediation analysis depicted separately for the undernourished group of children.

	Mediator				Dependent variables							
	Psychosocial stimulation (HOME)				Motor development (MoDQ)				Mental development (MeDQ)			
Antecedents	*B*	SE	*t*	*R* ^2^	*B*	SE	*t*	*R* ^2^	*B*	SE	*t*	*R* ^2^
				0.75				0.13				0.11
Constant	–3.45	7.37	–0.47		69.79	48.74	1.43		29.10	32.71	0.89	
Psychosocial stimulation	--	--	--		–2.33	4.05	–0.58		0.35	0.38	0.91	
Nutritional status (MUAC)	1.88	0.59	3.19***		1.64	0.57	2.90		2.61	2.72	0.96	

					Effect	SE	LLCI	ULCI	Effect	SE	LLCI	ULCI
Indirect effects of Nutritional status of the child (MUAC) via psychosocial stimulation					3.09	1.28	0.94	5.97	0.65	0.68	–0.55	2.20

					Effect	SE	LLCI	ULCI	Effect	SE	LLCI	ULCI
Direct effects of Nutritional status of the child (MUAC)					–2.33	4.05	–10.34	5.68	2.61	2.72	–2.76	7.98

LLCI, bias corrected lower limit confidence interval; ULCI, bias corrected upper limit confidence interval. Unstandardized regression coefficients are shown; bootstrap sample size is 5000. Significance levels have been denoted as- * Significant at *p <* 0.05 level, ** Significant at *p <* 0.01 level, *** Significant at *p <* 0.001 level. MUAC, mid-upper arm circumference measure, HOME total- measure of psychosocial stimulation; MoDQ, motor development quotient; MeDQ, mental development quotient.

**Table 8:** Regression table for mediation analysis with individual domains of psychosocial stimulation.

Domains of the mediator in the model (psychosocial stimulation)	Dependent measure (MoDQ/MeDQ)	Effect of IV on mediator (Path a)	Unique effect of mediator (Path b)	Indirect effect of IV on DV via M (Path ab)	BC 95% CI	
					Lower	Upper
1. Home environment	Motor development (MoDQ)	0.27 (0.13)*	1.38 (0.79)	0.38 (0.27)	–0.07	1.00
	Mental development (MeDQ)		1.01 (0.51)*	0.27 (0.19)	0.01	0.72
2. Language and play	Motor development (MoDQ)	0.11 (0.08)	3.75 (1.17)***	0.40 (0.34)	–0.25	1.15
	Mental development (MeDQ)		1.73 (0.76)**	0.18 (0.17)	–0.09	0.56
3. Mother’s affection and affirmation	Motor development (MoDQ)	0.20 (0.08)**	3.40 (1.29)**	0.69 (0.36)	0.08	1.51
	Mental development (MeDQ)		2.26 (0.83)**	0.46 (0.26)	0.05	1.07
4. Travel and exploration	Motor development (MoDQ)	–0.02 (0.09)	5.60 (1.12)***	–0.09 (0.44)	–0.04	0.76
	Mental development (MeDQ)		1.64 (0.74)**	–0.03 (0.15)	–0.36	0.23

All coefficients reported for paths a,b, and ab are unstandardized slopes with the corresponding standard error of the slope in parentheses. Bias-corrected CI of each indirect effect is based on 5,000 resamples. Significance levels have been denoted as- * Significant at *p <* 0.05 level, ** Significant at *p <* 0.01 level, *** Significant at *p <* 0.001 level. Paths a, b, ab correspond to the depicted mediation model (Figure 1).

### Exploratory analysis

While the mediation analysis was performed substituting the total score of psychosocial stimulation as the mediator in the model, it was also our concern to explore as to which domain of psychosocial stimulation plays a determining role in predicting the developmental outcomes. To ascertain which domain of psychosocial stimulation had more influence on child development, the simple mediation analysis was repeated by substituting each domain as the mediator separately. The four domains of psychosocial stimulation are- home environment, language and play, mother’s affection and affirmation, travel and exploration. The domain of home environment fully mediated in significantly predicting the mental development among children [Effect = 0.27, 95% C.I. (0.01, 0.72)]. The domain of the mother’s affection and affirmation fully mediated in significantly predicting the motor [Effect = 0.69, 95% C.I. (0.08, 1.51)] as well as mental development [Effect = 0.46, 95% C.I. (0.05, 1.07)] in children. The mediational influence of the other two domains- language and play, travel and exploration on developmental outcomes were not found significant.

The study further explores if the mediational influences of psychosocial stimulations hold true on the groups of healthy and undernourished children separately as well. The total sample (*N* = 275) was split into two groups of healthy (*N* = 103) and undernourished children (*N* = 172). The simple mediation analysis (model 4) was repeated on each group with nutritional status (MUAC) predicting developmental outcomes (MoDQ and MeDQ) when psychosocial stimulation (HOME) mediates the association. The family’s annual income, parental educational status, father’s occupation, and the child’s residence were treated as covariates in the model. In the case of healthy children, the indirect effects of nutritional status on motor development [Effect = 0.25, 95% C.I. (−1.56, 3.18)] and mental development [Effect = 0.34, 95% C.I. (−1.08, 2.48)] were not found statistically significant. In the case of undernourished children, the indirect effects of nutritional status on motor development were found statistically significant [Effect = 3.09, 95% C.I. (0.94, 5.97)]. However, the mediational effects were not found statistically significant for mental development [Effect = 0.65, 95% C.I. (−0.55, 2.20)].

Table 9 shows the comparison in motor and mental development between the experimental and control group at baseline before intervention. Table 10 shows the results of paired-samples *t*-test conducted to compare changes in the motor development (MoDQ) and mental development (MeDQ) of the undernourished children in the intervention and no intervention condition. Results reveal that after a duration of 25-weeks, there was a significant difference in the motor development of the participants in the *experimental group* when compared with the baseline assessment before the intervention (*M =* 87.26*, SD =* 17.18) and the post-test assessment (*M =* 90.49*, SD =* 12.05) after intervention; *t*(81) = 2.568, *p =* 0.012. The effect size is large (Cohen’s *d =* 1.086). Similarly, a significant difference was found in the mental development of the participants in the *experimental group* when compared with the baseline assessment before the intervention (*M =* 80.34*, SD =* 14.21) and the post-test assessment (*M =* 85.67*, SD =* 12.13) after intervention; *t*(81) = 4.506, *p =* 0.001. The effect size is large (Cohen’s *d =* 1.082). The results suggest that psychosocial stimulation intervention enhances the motor and mental development of undernourished children. Specifically, the results indicate that when the undernourished child receives psychosocial stimulation through positive parenting practices and nurturance, they demonstrate better motor and mental development than undernourished children who receive food-based intervention alone.

**Table 9:** Independent measures *t-*test results comparing the baseline conditions before intervention between the experimental and the control group children.

Measure	Experimental		Control group		*t* (142)	*p*	Cohen’s *d*
	*M*	*SD*	*M*	*SD*			
Total (*N* = 144)	*N* = 83		*N* = 61				
Motor development (MoDQ)	87.26	17.18	91.39	15.97	1.468	0.144	0.248
Mental development (MeDQ)	80.34	14.21	83.32	9.77	1.409	0.161	1.039

**Table 10:** Repeated measures *t-*test results comparing the pre-test and the post-test assessment to test for the effectiveness of the intervention program.

Measure	Pre-test		Post-test		*t*	*p*	Cohen’s *d*
	*M*	SD	*M*	SD			
Experimental group (*N* = 83)							
Motor development (MoDQ)	87.26	17.18	90.49	12.05	2.568	0.012	1.086
Mental development (MeDQ)	80.34	14.21	85.67	12.13	4.506	0.000	1.082
Control group (*N* = 61)							
Motor development (MoDQ)	89.39	17.97	87.91	12.65	1.611	0.112	1.201
Mental development (MeDQ)	83.32	9.77	82.24	14.23	0.712	0.479	1.246

## Discussion

The present study examined whether psychosocial stimulation explains the association between the nutritional status and the developmental outcomes in the pre-school children. We found that the psychosocial stimulation fully mediated in significantly predicting the motor and mental development of children after controlling for parental education status, father’s occupation, family income, and the place of residence.

We explored psychosocial stimulation in the context of the home environment of the child. The quality and quantity of psychosocial stimulation received by the child are contingent upon his/her interaction with the resources available in the surrounding home environment. It may be reasoned that a healthy child receives more psychosocial stimulation from his/her active interactions with the people and the surrounding resources. On the contrary, an undernourished child who is rather passive and inactive misses out on these stimulations. The environment of the child whether it is home or school acts as a key source of psychosocial stimulation (Riyadi et al., 2019).

The results of the study also indicate that the educational levels of parents as well as the income status of the family significantly predicted the availability of psychosocial stimulation to the growing child. Other researchers also report that the place of residence, educational qualifications of the parents as well as their economic standards determine the quality of parenting practices responsible for the development of the child (Petterson and Albers, 2001). An understandable reason being that the psychosocial stimulations find due regard among the homes of educated and socially aware parents who are attentive, devote more time, and put in extra efforts in nurturing the child (Conway et al., 2019). These are also the sources of psychosocial stimulation to the child. Hence, the present study assessed the mediational role of psychosocial stimulation while covarying the parental educational status, father’s occupation, family income, and place of residence within the tested model.

The present study establishes that psychosocial stimulations are the mediating factors that govern how nutrition translates into effective child development. Findings from other studies also establish that coupling psychosocial stimulation along with nutritional supplementation could help buffer against the long-term damaging effects of undernutrition (Zvara et al., 2019). The domain describing the mother’s displays of affection and affirmation to the child also significantly mediated both motor and mental development. Therefore, we infer that playing with the child, cuddling, laughing, expressions of warmth, spending quality time, allowing the child to explore on his/her own, etc. as accounted for in the present study play a key role in predicting child development. Such practices are cost effective and have been implemented by other researchers effectively in the rural settings (Riyadi et al., 2019). These findings are supported in other studies that effective parenting practices during the early years have a positive influence on the child’s cognitive and socio-emotional development later in life as well (Walker et al., 2005; Dahl et al., 2017; Knauer et al., 2019). Including psychosocial stimulation-based intervention early in life has shown positive impact on the cognitive and socio-emotional development at the age of 31-years as well (Walker et al., 2022). On the contrary, a chaotic household adversely impacts a child’s early executive function skills as validated through teacher ratings and performance-based measures of the child’s behavioral regulation (Vernon-Feagans et al., 2016).

Analyzing for the mediational role of psychosocial stimulation among the healthy group and the undernourished group of children separately, significant mediation was reported only in the prediction of motor development among the undernourished group. One of the reasons why the mediational influence of psychosocial stimulation failed to sustain could be the decrease in the sample size when divided separately into two groups of healthy and undernourished children. Further, the MUAC measurements indicating the nutritional status among the healthy children ranged from 13.00 to 15.00 and in the case of undernourished children ranged between 11.20 and 13.00 (Table 3). Since MUAC is the predictor in the mediation model, the power of prediction is likely to be affected by the low range offered by MUAC as well as the decrease in the sample size to execute the operation. Further researches could benefit from working with larger samples as well as the inclusion of other measures to assess the nutritional status of children.

The present study is also an important theoretical contribution to the field of child development. The model of child development proposed in the study ascertains that psychosocial stimulation in synchronization with nutrition is a pre-requisite for every growing child. The model finds its significance, particularly in mitigating the aftermaths of undernutrition in the race of development throughout life (Walker et al., 2022). The Study by Burger (2010) in lending support to the present findings also alerts us that the developmental aftermaths of unfavorable learning conditions in preschool children, mainly from socio-economically disadvantaged backgrounds, cannot be compensated entirely through interventions once they reach schools. Therefore, the significance of the preschool stage, particularly in view of the design and implementation of intervention programs, needs to be considered seriously (Scrimshaw, 1998).

## Implications of the study

The present study addresses a pressing global concern of undernutrition and its aftermath on child development. It can be inferred that psychosocial stimulations imparted through positive parenting practices share a significant association with the child’s nutritional status as well as indices of child development. The study aims to draw the attention of the NGOs and policymakers and attempts to bring about a paradigm shift in their intervention approaches in dealing with the problem of undernutrition. The study is also significant from the aspect of understanding the role of parenting and the home environment in shaping the developmental course of every child. The findings emphasize the importance of spending quality time and smart parenting strategies particularly in the formative stages of the child’s developmental course. It may be a timely reminder particularly in the present times when more women are working and the children are being raised in day-care centers, nursery, or by a single parent. The theoretical contribution of the paper is found in the proposed model of holistic child development accounting for the interconnectedness of the biological, psychological, and social factors. The proposed model may have practical applications in predicting the child’s higher achievement at school, higher employment, and earnings, better health outcomes throughout life, less dependency on the external agencies for support as well as lower crime rates in the future (Engle et al., 2007).

## Limitations and future directions

The present study is limited in its scope and generalization for including the research participants only from a single location in India. Future studies with samples representing other lower-middle-income as well as developed countries would help to understand the bigger picture of the roles and interplays of psychosocial practices in predicting child development. A 25-week intervention program may not be sufficient to depict all the changes brought about by the psychosocial stimulation model. Pursuing longitudinal observations of the developmental course of the child in future studies may help in better predictions of child development. Being a field experiment, the study is limited by the possibility of confounds. The study is also limited due to the exclusion of children being raised by single parents which could have explained different dynamics of psychosocial stimulations as well as determinants of child development. Further, in including the children raised by both the parents in the present study, we did not account for the role of the father as a source of psychosocial stimulation different from that provided by the mother. Researchers in the future could design studies differentiating the types of psychosocial stimulations offered by both the parents and exploring their unique impressions on the child’s development. The interpersonal relationships between husband and wife is also a crucial determinant of the family environment, psychosocial stimulation, and eventually child development. Studies in the future when include children coming from chaotic and abusive circumstances could be useful in understanding the role of psychosocial stimulations further.

## Data availability statement

The datasets presented in this study can be found in online repositories. The names of the repository/repositories and accession number(s) can be found at: https://osf.io/2mvpt/files/.

## Ethics statement

The Institutional Human Ethics Review Board (IHERB) approved the present study. We obtained the written informed consent of the mother/primary caregiver of the child to administer the performance test of developmental delay (DASII).

## Author contributions

BD: Conceptualization, Data curation, Formal analysis, Investigation, Methodology, Project administration, Resources, Software, Validation, Visualization, Writing – original draft, Writing – review & editing. SK: Conceptualization, Data curation, Formal analysis, Investigation, Methodology, Project administration, Resources, Software, Supervision, Writing – review & editing.

## References

[ref1] Aboud F. E. Yousafzai A. K. (2015). Global Health and development in early childhood. Annu. Rev. Psychol. 66, 433–457. doi: 10.1146/annurev-psych-010814-015128, PMID: 25196276

[ref2] Achadi E. Ahuja A. Bendech M. A. Bhutta Z. A. De-Regil L. M. Fanzo J.. (2016). Global nutrition report: from Promise to impact – ending malnutrition by 2030. In International Food Policy Research. Available at: http://ebrary.ifpri.org

[ref3] Baron R. M. Kenny D. A. (1986). The moderator-mediator variable distinction in social psychological research. Conceptual, strategic, and statistical considerations. J. Pers. Soc. Psychol. 51, 1173–1182. doi: 10.1037/0022-3514.51.6.1173, PMID: 3806354

[ref4] Bayley N. (1969). Manual for the Bayley scales of infant development. New York: Psychological Corporation.

[ref5] Black R. E. Victora C. G. Walker S. P. Bhutta Z. A. Christian P. De Onis M.. (2013). Maternal and child undernutrition and overweight in low-income and middle-income countries. Lancet 382, 427–451. doi: 10.1016/S0140-6736(13)60937-X, PMID: 23746772

[ref6] Blössner M. De Onis M. Prüss-Üstün A. Campbell-Lendrum D. Corvalán C. Woodward A. (2005). Malnutrition quantifying the health impact at national and local levels World Health Organization nutrition for health and development protection of the human environment Geneva 2005 WHO library cataloguing-in-publication data. Environ. Burden Diseases 12, 1–51.

[ref7] Bradley R. H. (1993). Children’s home environments, health, behavior, and intervention efforts: a review using the HOME inventory as a marker measure. Genet. Soc. Gen. Psychol. Monogr. 119, 437–490. PMID: 8150270

[ref8] Bronfenbrenner U. Morris P. A. (2007). “The bioecological model of human development” in Handbook of Child Psychology (John Wiley & Sons, Inc.)

[ref9] Burger K. (2010). How does early childhood care and education affect cognitive development? An international review of the effects of early interventions for children from different social backgrounds. Early Child. Res. Q. 25, 140–165. doi: 10.1016/j.ecresq.2009.11.001

[ref10] Conway A. Waldfogel J. Wang Y. (2019). Disparities in kindergarteners’ executive functions at kindergarten entry: relations with parenting and child care. Early Child. Res. Q. 48, 267–283. doi: 10.1016/j.ecresq.2019.03.009, PMID: 36322600

[ref11] Dabar D. Das R. Nagesh S. Yadav V. Mangal A. (2016). A community-based study on growth and development of under-five children in an urbanized village of South Delhi. J. Trop. Pediatr. 62, 446–456. doi: 10.1093/tropej/fmw026, PMID: 27143343

[ref12] Dahl A. Satlof-Bedrick E. S. Hammond S. I. Drummond J. K. Waugh W. E. Brownell C. A. (2017). Explicit scaffolding increases simple helping in younger infants. Dev. Psychol. 53, 407–416. doi: 10.1037/dev0000244, PMID: 27854464 PMC5323366

[ref13] Dixit P. Gupta A. Dwivedi L. K. Coomar D. (2018). Impact evaluation of integrated child development Services in Rural India: propensity score matching analysis. SAGE Open 8. doi: 10.1177/2158244018785713

[ref14] Engle P. L. Black M. M. Behrman J. R. Cabral de Mello M. Gertler P. J. Kapiriri L.. (2007). Strategies to avoid the loss of developmental potential in more than 200 million children in the developing world. Lancet 369, 229–242. doi: 10.1016/S0140-6736(07)60112-3, PMID: 17240290

[ref15] Georgieff M. K. (2007). Nutrition and the developing brain: nutrient priorities and measurement. Am. J. Clin. Nutr. 85, 614–620. doi: 10.1093/ajcn/85.2.614S 17284765

[ref16] Government of India. (2011). Operational guidelines on facility based Management of Children with SAM. Government of India.

[ref17] Grantham-McGregor S. Cheung Y. B. Cueto S. Glewwe P. Richter L. Strupp B. (2007). Developmental potential in the first 5 years for children in developing countries. Lancet 369, 60–70. doi: 10.1016/S0140-6736(07)60032-4, PMID: 17208643 PMC2270351

[ref18] Hattabi S. Bouallegue M. Mhenni T. Halouani J. Chtourou H. (2021). Effect of a plyometric training program on the physical parameters of ADHD children: behavioral and cognitive consequences. Int. J. Sport Stud. Health 4, 1–5. doi: 10.5812/intjssh.118756

[ref19] Hayes A. (2017). Introduction to mediation, moderation, and conditional process analysis. Available at: www.guilford.com/ebooks 10.1017/SJP.2021.46 35923144

[ref20] Hoddinott J. Maluccio J. A. Behrman J. R. Flores R. Martorell R. (2008). Effect of a nutrition intervention during early childhood on economic productivity in Guatemalan adults. Lancet 371, 411–416. doi: 10.1016/S0140-6736(08)60205-6, PMID: 18242415

[ref21] Knauer H. A. Ozer E. J. Dow W. H. Fernald L. C. H. (2019). Parenting quality at two developmental periods in early childhood and their association with child development. Early Child. Res. Q. 47, 396–404. doi: 10.1016/j.ecresq.2018.08.009, PMID: 35546925

[ref22] Kohli A. Mohanty M. Kaur K. L. (2005). Adaptation of a home inventory for children in simple Hindi. J. Indian Assoc. Child Adolesc. Ment. Health 1, 5–12.

[ref23] Krishnan V. (1967). Madhya Pradesh District gazetteers: Sagar. Available at: http://cslrepository.nvli.in/handle/123456789/3153

[ref24] Larson L. M. Martorell R. Bauer P. J. (2018). A path analysis of nutrition, stimulation, and child development among Young children in Bihar. India. Child Develop. 89, 1871–1886. doi: 10.1111/cdev.13057, PMID: 29529358 PMC6174960

[ref25] McGrath M. Schafer A. (2014). Integrating psychosocial support into nutrition programmes in West Africa during the Sahel food crisis. Intervention 12, 115–126. doi: 10.1097/WTF.0000000000000019

[ref26] Murray L. Fiori-Cowley A. Hooper R. Cooper P. (1996). The impact of postnatal depression and associated adversity on early mother-infant interactions and later infant outcome. Child Dev. 67, 2512–2526. doi: 10.2307/1131637, PMID: 9022253

[ref27] Nahar B. Hossain M. I. Hamadani J. D. Ahmed T. Huda S. N. Grantham-McGregor S. M.. (2012). Effects of a community-based approach of food and psychosocial stimulation on growth and development of severely malnourished children in Bangladesh: a randomised trial. Eur. J. Clin. Nutr. 66, 701–709. doi: 10.1038/ejcn.2012.13, PMID: 22353925

[ref28] NFHS. (2016). National family health Survey-4. International Institute of Population Sciences (IIPS). Mumbai: Ministry of Health and Family Welfare. Available at: https://www.google.com/search?rlz=1C1CHBD_enIN804IN804&ei=UA9qXpW2LMWm9QOb5LjAAg&q=NFHS.+%282016%29.+National+Family+Health+Survey-4.+International+Institute+of+Population+Sciences+%28IIPS%29.+Mumbai%3A+Ministry+of+Health+and+Family+Welfare&oq=NFHS.+%2820

[ref29] Pathak P. (1970). Mental and motor growth of Indian babies: 1–30 months. Final report. Department of Child Development, Faculty of Home Science, University of Baroda, Baroda. Available at: https://eric.ed.gov/?id=ED094870

[ref30] Petterson S. M. Albers A. B. (2001). Effects of poverty and maternal depression on early child development. Child Dev. 72, 1794–1813. doi: 10.1111/1467-8624.00379, PMID: 11768146

[ref31] Phatak P. (1997). Manual for using developmental assessment scales for Indian infants (DASII); based on revised Baroda norms. Pune, India: Anand Agencies.

[ref32] Rahman A. Iqbal Z. Bunn J. Lovel H. Harrington R. (2004). Impact of maternal depression on infant nutritional status and illness: a cohort study. Arch. Gen. Psychiatry 61, 946–952. doi: 10.1001/archpsyc.61.9.946, PMID: 15351773

[ref33] Raver C. Knitze J. (2002). Ready to enter: what research tells policymakers about strategies to promote social and emotional school readiness among three- and four-year-old children. Promoting the Emotional Well-Being of Children and Families. Available at: https://academiccommons.columbia.edu/doi/10.7916/D82V2QVX

[ref34] Riyadi H. Khomsan A. Anwar F. Herawati T. Hernawati N. Rahma A.. (2019). Nutrition education and psychosocial stimulation improves child development in rural early childhood education in Indonesia. J. Food Nutr. Res. 7, 717–724. doi: 10.12691/jfnr-7-10-5

[ref35] Ruel M. T. Alderman H. (2013). Nutrition-sensitive interventions and programmes: how can they help to accelerate progress in improving maternal and child nutrition? Lancet 382, 536–551. doi: 10.1016/S0140-6736(13)60843-0, PMID: 23746780

[ref36] Scrimshaw N. (1998). Malnutrition, brain development, learning, and behavior. Nutr. Res. 18, 351–379. doi: 10.1016/S0271-5317(98)00027-X, PMID: 34767796

[ref37] Selvam S. Thomas T. Shetty P. Thennarasu K. Raman V. Khanna D.. (2018). Development of norms for executive functions in typically-developing Indian urban preschool children and its association with nutritional status. Child Neuropsychol. 24, 226–246. doi: 10.1080/09297049.2016.1254761 27907279

[ref38] Tajari S. N. Gholami S. Rostami R. Trabelsi K. Taheri M. (2023). The effect of perceptual-motor exercise on temporal dynamics of cognitive inhibition control in children with developmental coordination disorder. Ment. Health Phys. Act. 24:100495. doi: 10.1016/j.mhpa.2022.100495

[ref39] UNHCR. (2017). The sustainable development goals and addressing statelessness. In UN High Commissioner for Refugees (UNHCR). Available at: https://www.refworld.org/docid/58b6e3364.html

[ref40] UNICEF (2007). The state of World’s children 2008: child survival. J. Tropical Pediatrics 57. doi: 10.1093/tropej/fmr064

[ref41] UNICEF. (2012). Integrating early childhood development (ECD) activities into nutrition Programmes in emergencies. Why, What and How. UNICEF.

[ref42] Vernon-Feagans L. Willoughby M. Garrett-Peters P. (2016). Predictors of behavioral regulation in kindergarten: household chaos, parenting, and early executive functions. Dev. Psychol. 52, 430–441. doi: 10.1037/dev0000087, PMID: 26751500 PMC4760868

[ref43] Walker S. Chang S. Powell C. Grantham-McGregor S. (2005). Effects of early childhood psychosocial stimulation and nutritional supplementation on cognition and education in growth-stunted Jamaican children: prospective. Lancet 366, 1804–1807. doi: 10.1016/S0140-6736(05)67574-5, PMID: 16298218

[ref44] Walker S. P. Chang S. M. Wright A. S. Pinto R. Heckman J. J. Grantham-McGregor S. M. (2022). Cognitive, psychosocial, and behaviour gains at age 31 years from the Jamaica early childhood stimulation trial. J. Child Psychol. Psychiatry 63, 626–635. doi: 10.1111/jcpp.13499 34403137 PMC8850528

[ref45] Walker S. P. Powell C. A. Grantham-McGregor S. M. Himes J. H. Chang S. M. (1991). Nutritional supplementation, psychosocial stimulation, and growth of stunted children: the Jamaican study. Am. J. Clin. Nutr. 54, 642–648. doi: 10.1093/ajcn/54.4.642, PMID: 1897471

[ref46] Walker S. P. Wachs T. D. Meeks Gardner J. Lozoff B. Wasserman G. A. Pollitt E.. (2007). Child development: risk factors for adverse outcomes in developing countries. Lancet 369, 145–157. doi: 10.1016/S0140-6736(07)60076-2, PMID: 17223478

[ref47] WHO. (2013). Global nutrition policy review: what does it take to scale up nutrition action?. Available at: http://apps.who.int/iris/handle/10665/84408

[ref48] WHO, UNICEF. (2009). WHO child growth standards and identification in infants. In WHO Library. Available at: http://www.who.int/nutrition/publications/severemalnutrition/9789241598163/en/

[ref49] Zvara B. J. Keim S. A. Boone K. M. Anderson S. E. (2019). Associations between parenting behavior and executive function among preschool-aged children born very preterm. Early Child. Res. Q. 48, 317–324. doi: 10.1016/j.ecresq.2019.01.012, PMID: 32189828 PMC7079770

